# Impact of the timing of metformin administration on glycaemic and glucagon-like peptide-1 responses to intraduodenal glucose infusion in type 2 diabetes: a double-blind, randomised, placebo-controlled, crossover study

**DOI:** 10.1007/s00125-024-06131-6

**Published:** 2024-04-01

**Authors:** Cong Xie, Peter Iroga, Michelle J. Bound, Jacqueline Grivell, Weikun Huang, Karen L. Jones, Michael Horowitz, Christopher K. Rayner, Tongzhi Wu

**Affiliations:** 1 Adelaide Medical School and Centre of Research Excellence in Translating Nutritional Science to Good Health, The University of Adelaide, Adelaide, Australia; 2 Endocrine and Metabolic Unit, Royal Adelaide Hospital, Adelaide, Australia; 3 Department of Gastroenterology and Hepatology, Royal Adelaide Hospital, Adelaide, Australia

**Keywords:** Glucagon-like peptide-1, Insulin, Metformin, Postprandial glycaemia, Preload, Type 2 diabetes

## Abstract

**Aims/hypothesis:**

Metformin lowers postprandial glycaemic excursions in individuals with type 2 diabetes by modulating gastrointestinal function, including the stimulation of glucagon-like peptide-1 (GLP-1). The impact of varying the timing of metformin administration on postprandial glucose metabolism is poorly defined. We evaluated the effects of metformin, administered at different intervals before an intraduodenal glucose infusion, on the subsequent glycaemic, insulinaemic and GLP-1 responses in metformin-treated type 2 diabetes.

**Methods:**

Sixteen participants with type 2 diabetes that was relatively well-controlled by metformin monotherapy were studied on four separate days in a crossover design. On each day, participants were randomised to receive a bolus infusion of metformin (1000 mg in 50 ml 0.9% saline) via a nasoduodenal catheter at *t* = −60, −30 or 0 min (and saline at the other timepoints) or saline at all timepoints (control), followed by an intraduodenal glucose infusion of 12.56 kJ/min (3 kcal/min) at *t* = 0–60 min. The treatments were blinded to both participants and investigators involved in the study procedures. Plasma glucose, insulin and total GLP-1 levels were measured every 30 min between *t* = −60 min and *t* = 120 min.

**Results:**

There was a treatment-by-time interaction for metformin in reducing plasma glucose levels and increasing plasma GLP-1 and insulin levels (*p*<0.05 for each). The reduction in plasma glucose levels was greater when metformin was administered at *t* = −60 or −30 min vs *t* = 0 min (*p*<0.05 for each), and the increases in plasma GLP-1 levels were evident only when metformin was administered at *t* = −60 or −30 min (*p*<0.05 for each). Although metformin did not influence insulin sensitivity, it enhanced glucose-induced insulin secretion (*p*<0.05), and the increases in plasma insulin levels were comparable on the 3 days when metformin was given.

**Conclusions/interpretation:**

In well-controlled metformin-treated type 2 diabetes, glucose-lowering by metformin is greater when it is given before, rather than with, enteral glucose, and this is associated with a greater GLP-1 response. These observations suggest that administration of metformin before meals may optimise its effect in improving postprandial glycaemic control.

**Trial registration:**

www.anzctr.org.au ACTRN12621000878875

**Funding:**

The study was not funded by a specific research grant.

**Graphical Abstract:**

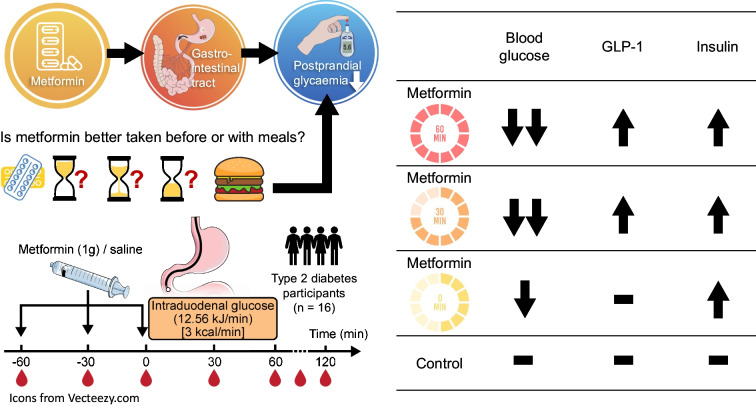


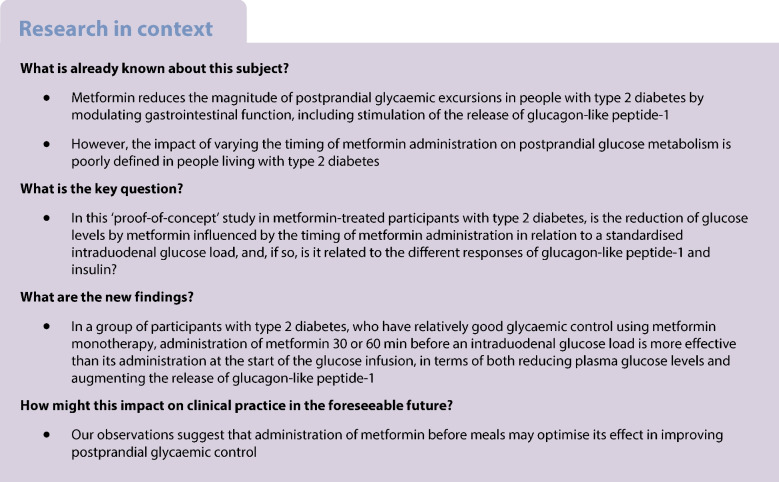


## Introduction

Metformin remains the recommended first-line pharmacotherapy for type 2 diabetes in most clinical guidelines, but its mode of action remains incompletely understood. An improved understanding of the mechanisms underlying glucose-lowering by metformin would provide the potential to refine its clinical application in the management of type 2 diabetes.

While a number of mechanistic studies have found that metformin lowers fasting blood glucose levels by suppressing hepatic glucose production [1–3], a substantial body of in vivo data suggest that much of the glucose-lowering action occurs at the level of the gastrointestinal tract, irrespective of its systemic bioavailability [3, 4]. In line with this concept, administration of metformin by the enteral route was found to be more effective in glucose-lowering than intravenous or intraportal administration [5]. Moreover, a delayed-release formulation of metformin with minimal systemic exposure was shown to be as effective as immediate- or extended-release formulations [6, 7]. Preclinical and clinical studies have uncovered several gastrointestinal effects of metformin, including stimulation of the incretin hormone glucagon-like peptide-1 (GLP-1) [8], slowing of gastric emptying [8], suppression of intestinal glucose absorption [9], inhibition of bile acid resorption [10, 11] and modulation of the gut microbiota [12]. The findings that blockade of GLP-1 signalling abolished metformin-induced suppression of hepatic glucose output in rodents [13], and markedly attenuated the effect of metformin in reducing the glycaemic response to a mixed meal in people with type 2 diabetes [14], attest to a major role for GLP-1 in mediating the glucose-lowering effect of metformin. GLP-1 modulates glucose homeostasis via pleiotropic actions [15, 16], including stimulation of insulin secretion, suppression of glucagon release [17], slowing of gastric emptying [18] and inhibition of appetite [19].

There is considerable evidence that strategies designed to lower postprandial glycaemia in type 2 diabetes by boosting GLP-1 secretion are preferably delivered before a meal [20–22]. For example, a ‘preload’ of whey protein consumed 30 min before a potato meal in individuals with type 2 diabetes was more effective in stimulating GLP-1 secretion, slowing gastric emptying and reducing the postprandial glycaemic excursion, when compared to its consumption with the meal [20]. In another study, a small amount of whey preload was shown to be sufficient to reduce postprandial glycaemia and augment glucose-lowering by the dipeptidyl peptidase-4 inhibitor vildagliptin in type 2 diabetes [21]. Routine advice is to ingest metformin with meals, with the rationale that this approach will minimise potential gastrointestinal adverse effects; however, this has not been shown to be the case [23]. The impact on postprandial glucose-lowering of altering the timing of metformin administration in relation to meals has been poorly characterised, although an open-label pilot study in five metformin-treated type 2 diabetes patients was indicative of improved glucose-lowering, enhanced GLP-1 secretion and slowed gastric emptying when metformin (1000 mg) was administered 30 min before the meal rather than with the meal [24].

We hypothesised that varying the timing of metformin administration before a standardised nutrient load would affect glucose-lowering, and, in this ‘proof-of-concept’ study, we evaluate the effects of metformin administered by intraduodenal perfusion at different time intervals (0, 30 or 60 min) before a standardised intraduodenal glucose load on plasma glucose, GLP-1 and insulin levels in metformin-treated participants with type 2 diabetes.

## Methods

### Participants

Nineteen participants with type 2 diabetes, managed by a stable dose of metformin monotherapy (500–2000 mg daily for at least 3 months), were recruited from the community and provided written informed consent. Two participants withdrew due to intolerance of the nasoduodenal catheter, and one withdrew for personal reasons prior to the first study visit. Accordingly, 16 participants with type 2 diabetes (race and ethnicity: 16 white [self-reported], sex and gender: 14 men and two women [self-reported], age: 69.9±1.9 years, BMI: 28.7±1.0 kg/m^2^, HbA_1c_: 48.2±1.6 mmol/mol [6.6±0.1%], known duration of diabetes: 10.4±2.6 years) completed the study and were included in the final analysis. No participant had significant gastrointestinal symptoms, impaired renal or liver function, a history of gastrointestinal surgery, or a requirement for medication that is known to affect gastrointestinal function or appetite. The protocol was approved by the Central Adelaide Local Health Network Human Research Ethics Committee (reference number: 2021/HRE00130), and prospectively registered in the Australian New Zealand Clinical Trials Registry (ACTRN12621000878875).

### Protocol

**Fig. 1:**
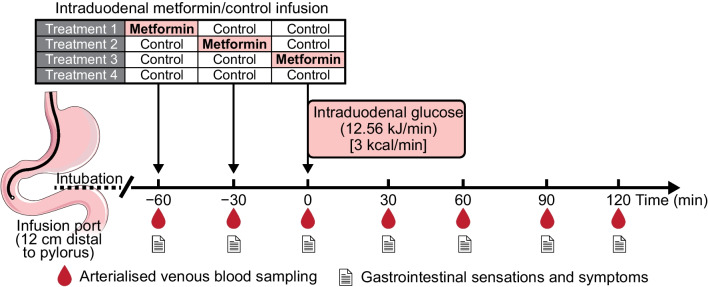
Study protocol

Each participant was studied at four different study visits, separated by at least 7 days, in a double-blind, randomised, crossover fashion (Fig. 1). During their involvement in the study, participants were instructed to maintain their usual dose of metformin, except for adjusting the time of dosing prior to each study visit. Specifically, participants treated with immediate-release metformin (*n*=11) withheld the morning dose on each study day, while participants taking extended-release metformin (*n*=5) withheld the evening dose the day before and the morning dose on each study day. Participants were asked to maintain their usual diet throughout and refrain from vigorous exercise and alcohol for 24 h before each study visit. On the evening before each study day (approximately 19:00h), participants consumed a standardised meal (beef lasagne, 2472 kJ, McCain Foods, Australia), and then fasted from solids and liquids other than water until the following morning, when they attended the Clinical Research Facility of the University of Adelaide at 08:00h.

On each study day, a silicone rubber catheter (Dentsleeve International, Canada) was inserted through an anaesthetised nostril into the stomach and allowed to pass into the small intestine by peristalsis. The catheter was positioned with the infusion port located in the duodenum (12 cm distal to the pylorus). Correct positioning of the catheter was monitored continuously by measurement of the transmucosal potential difference in the stomach (approximately −40 mV) and the duodenum (approximately 0 mV), as described previously [25, 26]. An intravenous cannula was then placed into a forearm vein, and the arm was kept warm with a heat pad to allow sampling of ‘arterialised’ blood. Subsequently, each participant received one of four treatments by intraduodenal infusion over 2 min in a double-blind, randomised fashion: (1) 1000 mg metformin (PCCA, Australia) dissolved in 50 ml 0.9% saline (154 mmol/l NaCl) at *t* = −60 min and 50 ml 0.9% saline at *t* = −30 and 0 min [Met (−60 min)]; (2) 1000 mg metformin at *t* = −30 min and 0.9% saline at *t* = −60 and 0 min [Met (−30 min)]; (3) 1000 mg metformin at *t* = 0 min and 0.9% saline at *t* = −60 and −30 min [Met (0 min)]; or (4) 0.9% saline at *t* = −60, −30 and 0 min (control). The randomisation schedule was generated by an online tool (https://www.randomizer.org/). A designated research officer, who was not involved in data collection and analysis, was authorised to have access to the randomisation schedule and be responsible for the preparation of the study solutions, such that both the participants and the investigators involved in the study procedures were blinded to the treatments. At *t* = 0 min, a glucose solution (45 g glucose dissolved in water to a total volume of 180 ml) was infused into the duodenum over 60 min (i.e. 3 ml/min; 12.56 kJ/min [3 kcal/min]). At *t* = 60 min, the catheter was removed, and participants were monitored until *t* = 120 min. Blood was sampled at *t* = −60 min (immediately before the commencement of the infusion) and every 30 min between *t* = −60 and *t* = 120 min for measurements of plasma glucose, total GLP-1 and insulin levels. Nausea and appetite sensations were evaluated at the same intervals using 100 mm visual analogue scales [27].

### Measurements of plasma glucose, total GLP-1 and insulin levels

Plasma glucose levels were measured using the glucose oxidase technique using a 2300 STAT PLUS analyser (YSI, USA). Plasma total GLP-1 levels were measured by radioimmunoassay using GLPIT-36HK (Millipore, USA), with a sensitivity of 3 pmol/l and intra- and interassay CVs of 7.9% and 12.9%. Plasma insulin levels were measured by ELISA immunoassay (catalogue no. 10-1113, Mercodia, Sweden), with a sensitivity of 6 pmol/l and intra- and interassay CVs of 2.1% and 11.1%.

### Statistical analysis

The integrated AUCs (iAUCs) for plasma glucose, insulin and total GLP-1 in response to intraduodenal glucose infusion between *t* = 0 min and *t* = 120 min were calculated by using the trapezoidal rule and subtracting the baseline area. Whole-body insulin sensitivity was assessed by the Matsuda index [28] using the formula:

$$\frac{10,000}{18\times G_0\times{6\times I}_0\times18\times G_{\mathrm{mean}\;0-120\text{min}}\times 6 \times {I_\mathrm{mean\;0-120\text{min}}}}$$

where *G*_0_ is the fasting plasma glucose level (mmol/l), *I*_0_ is the fasting plasma insulin level (pmol/l), *G*_mean 0–120min_ represents the mean plasma glucose levels between *t* = 0 min and *t* = 120 min, and *I*_mean 0–120min_ represents the mean plasma insulin levels between *t* = 0 min and *t* = 120 min. Plasma glucose, insulin and total GLP-1 levels during fasting (*t* = −60, −30 and 0 min) and their iAUCs_0–120min_, as well as the Matsuda index, were compared between the four study days using one-factor repeated-measures ANOVA. Plasma glucose, GLP-1 and insulin levels and the insulin/glucose ratio were also analysed using two-factor repeated-measures ANOVA, with treatment and time as factors. Post hoc comparisons, adjusted for multiple comparisons by Bonferroni’s correction, were performed if the ANOVAs showed a significant interaction between treatment and time. The study was initially powered to detect differences in small intestinal glucose absorption between metformin treatments and the control. However, due to logistic challenges in assessing this endpoint, we have focused primarily on the differences between treatments in terms of plasma glucose iAUC following intraduodenal glucose infusion. Based on data derived from our previous study [8], inclusion of 16 participants was calculated to provide at least 90% power (at α = 0.01 to allow for subgroup comparisons) to detect a 20% difference in glucose iAUC_0–120min_ between metformin treatments and the control. All analyses were performed using Prism 9.5 software (GraphPad, USA). Data are presented as means ± SEM. A *p* value <0.05 was considered statistically significant.

## Results

### Plasma glucose levels

**Fig. 2:**
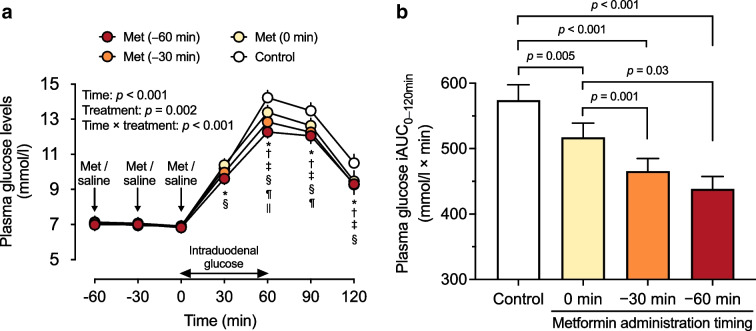
Effects of metformin (1000 mg) administered at −60 min [Met (−60 min)], −30 min [Met (−30 min)] or 0 min [Met (−0 min)] or 0.9% saline administered at *t* = −60, −30 and 0 min (control) on (**a**) plasma glucose levels and (**b**) the iAUC for plasma glucose between *t* = 0 min and *t* = 120 min (iAUC_0–120min_) in response to an intraduodenal glucose infusion at 12.56 kJ/min (3 kcal/min) between *t* = 0 min and *t* = 60 min in individuals with type 2 diabetes managed by metformin monotherapy (*n*=16). Repeated-measures ANOVA was used to determine statistical significance for differences in in plasma glucose levels between Met (−60 min), Met (−30 min), Met (0 min) and the control between *t* = −60 min and *t* = 120 min, with ANOVA results reported as *p* values for differences over time and by treatment and for treatment-by-time interactions. Post hoc comparisons were adjusted using Bonferroni’s correction: *p<0.05 for Met (−60 min) vs control; ^†^*p*<0.05 for Met (−30 min) vs control; ^‡^*p*<0.05 for Met (0 min) vs control; ^§^*p*<0.05 for Met (−60 min) vs Met (0 min); ^¶^*p*<0.05 for Met (−30 min) vs Met (0 min); ^||^ *p*<0.05 for Met (−60 min) vs Met (−30 min). One-way ANOVA was used to determine statistical significance for the difference in the plasma glucose iAUC_0–120min_ between treatments. Post hoc comparisons were adjusted using Bonferroni’s correction. Data are means ± SEM

Fasting plasma glucose levels at *t* = −60, −30 and 0 min did not differ between the four study days (Table 1). In response to intraduodenal glucose infusion (*t* = 0–60 min), plasma glucose levels increased promptly (time effect: *p*<0.001 for each; Fig. 2a). There was a treatment-by-time interaction between the four study days (*p*<0.001). Compared with the control, plasma glucose levels were lower between *t* = 30 min and *t* = 120 min after Met (−60 min), and between *t* = 60 min and *t* = 120 min after Met (−30 min) and Met (0 min). Compared with Met (0 min), plasma glucose levels were also lower between *t* = 30 min and *t* = 120 min after Met (−60 min) and between *t* = 60 min and *t* = 90 min after Met (−30 min). Moreover, plasma glucose levels were lower at *t* = 60 min after Met (−60 min) compared with Met (−30 min) (*p*<0.05 for each) (Fig. 2a). There was also a treatment effect on the plasma glucose iAUC_0–120min_ (*p*<0.001). Compared with the control, Met (−60 min), Met (−30 min) and Met (0 min) all reduced the plasma glucose iAUC_0–120min_ (*p*<0.01 for each), but the magnitude of this reduction was greater after Met (−60 min) and Met (−30 min) compared with Met (0 min) (*p*=0.03 and *p*=0.001, respectively). There was no difference in plasma glucose iAUC_0–120min_ between Met (−60 min) and Met (−30 min) (Fig. 2b).

**Table 1:** Plasma glucose, total GLP-1 and insulin levels during fasting and in response to an intraduodenal glucose infusion between *t* = 0 min and *t* = 60 min in participants with type 2 diabetes managed by metformin monotherapy

		Interventions				*p*_treatment_
	Time (min)	Met (−60 min)	Met (−30 min)	Met (0 min)	Control	
Glucose						
Plasma concentration (mmol/l)	−60	6.98±0.33	7.10±0.34	7.08±0.35	7.04±0.29	0.83
	−30	7.01±0.31	6.95±0.32	6.93±0.34	7.04±0.30	0.79
	0	6.84±0.31	6.91±0.33	6.84±0.33	6.88±0.28	0.91
iAUC_0–120min_ (mmol/l × min)		439±19.6	466±20.0	517±22.5	574±24.7	<0.001
GLP-1						
Plasma total concentration (pmol/l)	−60	14.88±0.99	15.71±1.15	15.45±0.91	15.21±0.83	0.57
	−30	15.73±1.21	15.28±1.36	14.87±1.01	14.92±0.98	0.74
	0	16.34±1.48	16.24±1.50	14.92±1.09	14.96±1.06	0.30
iAUC_0–120min_ (pmol/l × min)		1879±457	2025±380	1582±260	1338±241	0.078
Insulin						
Plasma concentration (pmol/l)	−60	36.49±3.81	38.74±5.24	41.29±5.52	40.24±5.71	0.48
	−30	40.35±4.63	39.49±5.96	35.03±4.80	41.63±4.63	0.23
	0	39.64±4.49	40.31±5.03	35.14±4.89	37.05±6.16	0.39
iAUC_0–120min_ (pmol/l × min)		20121±4098	21809±4264	21682±4291	15628±2403	0.008
Insulin/glucose ratio (pmol/mmol)	−60	5.29±0.57	5.49±0.73	5.87±0.79	5.73±0.76	0.55
	−30	5.75±0.62	5.73±0.86	4.97±0.60	5.90±0.64	0.21
	0	5.72±0.55	5.79±0.66	5.06±0.65	5.31±0.86	0.30
Matsuda index		5.32±0.65	5.41±0.79	6.16±0.97	6.37±0.95	0.38

Data are means ± SEM

*n*=16

Fasting measurements are at *t* = −60, −30 and 0 min

Glucose infusion was 12.56 kJ/min (3 kcal/min)

### Plasma total GLP-1 levels

**Fig. 3:**
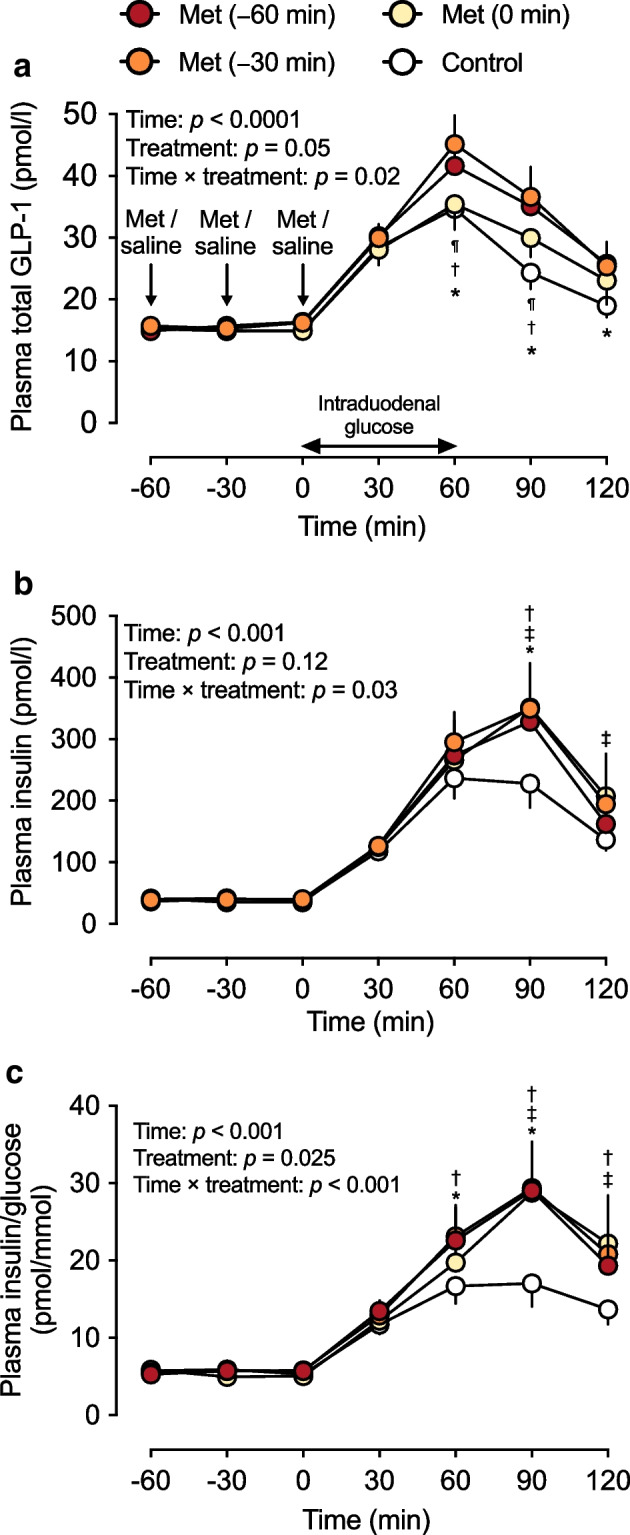
Effects of metformin (1000 mg) administered at −60 min [Met (−60 min)], −30 min [Met (−30 min)] or 0 min [Met (−0 min)] or 0.9% saline administered at *t* = −60, −30 and 0 min (control) on (**a**) plasma GLP-1 levels, (**b**) insulin levels and (**c**) insulin/glucose ratio in response to an intraduodenal glucose infusion at 12.56 kJ/min (3 kcal/min) between *t* = 0 min and *t* = 60 min in individuals with type 2 diabetes managed by metformin monotherapy (*n*=16). Repeated-measures ANOVA was used to determine statistical significance for differences between the four study days between *t* = −60 min and *t* = 120 min, with ANOVA results reported as *p* values for differences over time and by treatment and for treatment-by-time interactions. Post hoc comparisons were adjusted using Bonferroni’s correction: *p<0.05 for Met (−60 min) vs control; ^†^*p*<0.05 for Met (−30 min) vs control; ^‡^*p*<0.05 for Met (0 min) vs control; ^¶^ *p*<0.05 for Met (−30 min) vs Met (0 min). Data are means ± SEM

Fasting plasma total GLP-1 levels at *t* = −60, −30 and 0 min did not differ between the four study days (Table 1). In response to intraduodenal glucose infusion, plasma GLP-1 levels increased promptly on all study days (time effect: *p*<0.001 for each), peaking at *t* = 60 min before returning towards baseline. There was a treatment-by-time interaction for plasma total GLP-1 levels (*p*=0.02). Compared with the control, GLP-1 levels were higher between *t* = 60 min and *t* = 120 min after Met (−60 min) and between *t* = 60 min and *t* = 90 min after Met (−30 min). Compared with Met (0 min), GLP-1 levels were also higher between *t* = 60 min and *t* = 90 min after Met (−30 min) (*p*<0.05 for each) (Fig. 3a). There was also a non-significant tendency for metformin to augment plasma total GLP-1 iAUC_0–120min_ (*p*=0.078, Table 1).

### Plasma insulin levels, insulin/glucose ratio and insulin sensitivity

Fasting plasma insulin levels at *t* = −60, −30 and 0 min did not differ between the four study days (Table 1). In response to intraduodenal glucose infusion, plasma insulin levels increased promptly (time effect: *p*<0.001 for each), peaking at *t* = 60 min on the control day and at *t* = 90 min on the three metformin days before returning towards baseline. There was a treatment-by-time interaction on plasma insulin levels (*p*=0.03), with concentrations being higher at *t* = 90 min after Met (−60 min) and Met (−30 min) compared with the control, and between *t* = 90 min and *t* = 120 min after Met (0 min) compared with the control (*p*<0.05 for each) (Fig. 3b). There was also a treatment effect of metformin on the plasma insulin iAUC_0–120min_, which was greater on all the metformin days than the control day. However, there was no difference in the plasma insulin iAUC_0–120min_ between the metformin days (Table 1).

Similarly, the insulin/glucose ratio did not differ prior to intraduodenal glucose infusion between the four study days (Table 1), but increased substantially in response to intraduodenal glucose infusion, with a treatment effect (*p*=0.025) and a treatment-by-time interaction (*p*<0.001) between the study days. Compared with the control, the ratio was higher between *t* = 60 min and *t* = 90 min after Met (−60 min), between *t* = 60 min and *t* = 120 min after Met (−30 min), and between *t* = 90 min and *t* = 120 min after Met (0 min) (*p*<0.05 for each) (Fig. 3c).

The Matsuda index did not differ between the four study days (Table 1).

### Nausea and appetite

**Fig. 4:**
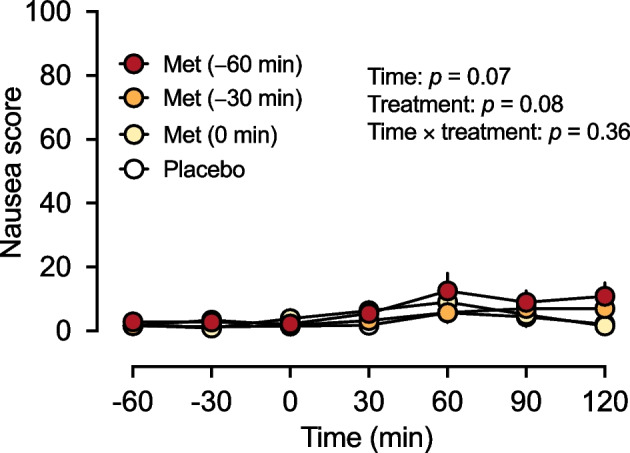
Effects of metformin (1000 mg) administered at −60 min [Met (−60 min)], −30 min [Met (−30 min)] or 0 min [Met (−0 min)] or 0.9% saline administered at *t* = −60, −30 and 0 min (control) on score for nausea before and during an intraduodenal glucose infusion at 12.56 kJ/min (3 kcal/min) between *t* = 0 min and *t* = 60 min in individuals with type 2 diabetes managed by metformin monotherapy (*n*=16). Repeated-measures ANOVA was used to determine statistical significance for differences between the four study days between *t* = −60 min and *t* = 120 min, with ANOVA results are reported as *p* values for differences over time and by treatment and for treatment-by-time interactions. Data are means ± SEM

The score for nausea was low and unchanged throughout the study, with no difference between the four treatments (Fig. 4). Appetite sensations (including hunger, fullness, desire to eat and anticipated meal size) were minimally altered before and after intraduodenal glucose infusion on all study days, with no difference between the four treatments (data not shown).

## Discussion

This study shows that administration of metformin 30 and 60 min before an intraduodenal glucose load is more effective than its administration at the start of the glucose infusion in terms of both reducing glycaemia and augmenting GLP-1 release in participants with metformin-treated type 2 diabetes. Moreover, earlier metformin administration did not induce gastrointestinal symptoms in this group of participants. These observations suggest that the timing of metformin administration can potentially substantially influence its efficacy for postprandial glucose-lowering, and that the current recommendation (at least for the immediate-release preparation) that people with type 2 diabetes should take metformin with meals may compromise its benefits.

In the current study, the exposure of the small intestine to metformin at different time intervals before the nutrient load was precisely controlled by direct infusion via a nasoduodenal catheter. This model circumvented the confounding impact of gastric emptying, which exhibits a wide inter-individual variation in both healthy individuals and those with type 2 diabetes [29–31]. For the same reason, the glucose solution was infused directly into the duodenum at a standardised rate (12.56 kJ/min [3 kcal/min]) within the physiological caloric range of gastric emptying [29]. These methods allowed a highly accurate assessment of the impact of the timing of small intestinal exposure to metformin on the subsequent glycaemic, insulinaemic and GLP-1 responses. We studied participants with type 2 diabetes who were already using a stable dose of metformin in order to optimise the translational relevance of our findings.

As in our previous study [8], acute dosing with metformin (1000 mg) did not affect fasting glucose concentrations over 60 min, but did reduce the glycaemic response to the intraduodenal glucose load in type 2 diabetes. Consistent with a pilot study in a small group of individuals with type 2 diabetes (*n*=5) [24], we showed that the reduction in the glycaemic response to intraduodenal glucose was greater after Met (−60 min) and Met (−30 min) than after Met (0 min). Although the plasma glucose iAUC_0–120min_ did not differ between Met (−60 min) and Met (−30 min), plasma glucose levels at the conclusion of intraduodenal glucose infusion (i.e. *t* = 60 min) were modestly lower after Met (−60 min). These observations provide compelling evidence to support the concept that a ‘preload’ of metformin before a meal has the potential to enhance its efficacy in postprandial glucose-lowering. Routine advice is to ingest metformin with meals in order to minimise potential gastrointestinal adverse effects despite the lack of an evidence base. However, our observations suggest that, at least in individuals with type 2 diabetes who are tolerant of metformin, such a practice may compromise the efficacy of metformin in reducing postprandial hyperglycaemia. If so, this is likely to have significant implications for the management of type 2 diabetes and the associated risk of microvascular complications, as postprandial glycaemia is the major determinant of overall glycaemic control in individuals with type 2 diabetes who have relatively good overall glycaemic control (HbA_1c_ of <64 mmol/mol [8.0%]) [32] and an independent predictor of cardiovascular disease and mortality risk in type 2 diabetes [33].

Metformin is known to exert multiple gastrointestinal actions, including stimulation of GLP-1 secretion [16], to improve postprandial glucose metabolism. Indeed, the greater reduction in the blood glucose response to intraduodenal glucose after Met (−60 min) and Met (−30 min) compared with Met (0 min) was accompanied by higher plasma total GLP-1 levels. The finding that the increase in GLP-1 was only evident after intraduodenal glucose infusion and was minimal when metformin was administered immediately before intraduodenal glucose suggests that the effect of metformin on GLP-1 is primarily related to modulation of the glucose–gut interaction [8, 34]. Although previous ex vivo studies employing NCI-H716 cells (a human L cell line) [35] or human ileal and colonic tissues [14] were indicative of a direct effect of metformin in stimulating GLP-1 release, clinical evidence suggests that the augmentation of postprandial GLP-1 secretion by metformin is largely indirect. For example, administration of metformin (1000 mg) via a nasoluminal catheter into the ileum, a region of the gut that is rich in L cells did not elicit any GLP-1 response over a 1 h period in patients with type 2 diabetes [8]. When 1500 mg metformin or placebo was given with a mixed liquid meal in participants with well-controlled type 2 diabetes (mean HbA_1c_ 48 mmol/mol [6.5%]), the overall postprandial GLP-1 response was greater with metformin than placebo, but this increase was evident approximately 2 h after the meal [14]. The mechanisms underlying an augmented GLP-1 response to intraduodenal glucose after Met (−60 min) and Met (−30 min) remain uncertain, but may reflect an effect of metformin on inhibiting glucose absorption in the upper small intestine, with consequent increased exposure of L cells to glucose more distally in the gut. In rats fed a high-fat-diet, metformin (200 mg/kg) was shown to reduce glucose absorption by inhibiting the expression of sodium glucose cotransporter-1 in the upper small intestine [36]. In mice fed a high-fat diet, metformin (400 mg/kg) also inhibited glucose uptake from the lumen into the circulation [37]. In individuals with type 2 diabetes, we observed that metformin (850 mg) ingested 30 min before an intraduodenal glucose infusion of 8.37 kJ/min (2 kcal/min) reduced the rate of glucose absorption by approximately 10%, and that the augmentation of GLP-1 secretion by metformin was related directly to the magnitude of inhibition of glucose absorption [9]. Accordingly, the varying effects of the timing of metformin administration may reflect the time intervals required for metformin to modulate the expression or function of glucose transporters in the upper small intestine. Metformin is also known to inhibit intestinal bile acid absorption [11] and modulate the composition of the gut microbiota [38], although neither is likely to be of relevance to the observations in the present study as glucose is a weak stimulus for gallbladder emptying and the current study was only conducted in an acute setting.

Plasma insulin levels were not affected by metformin during fasting, but were comparably augmented following intraduodenal glucose infusion on all the three metformin study days. The augmented insulin secretion may account for glucose-lowering by metformin in the current study, but the underlying mechanisms remain unclear. Although GLP-1 has the capacity to drive glucose-dependent stimulation of insulin secretion, the increase in GLP-1 was only evident after Met (−60 min) and Met (−30 min). Accordingly, other pathways are likely to be involved. Ex vivo studies using isolated human islets indicated that metformin is capable of augmenting insulin secretion at high glucose concentrations (16.6 mmol/l) [34]. However, our findings contrast with those of other clinical studies, in which acute administration of metformin was reported to have little effect on the insulin response to a standardised test meal in individuals with type 2 diabetes [8, 14, 24]. This discrepancy may be attributable to the slowing of gastric emptying induced by metformin [8], which would blunt the postprandial insulin response. The differences in both the doses and formulations of metformin may also be of relevance. In the current study, metformin was dissolved in saline for direct infusion into the small intestine, from where it would be rapidly absorbed. By contrast, oral administration of metformin requires a period of time for a tablet to break down and enter the small intestine. Whole-body insulin sensitivity (assessed by the Matsuda index) did not differ between the four study days. This is consistent with the outcomes of previous studies in people with type 2 diabetes, in which metformin, even after sustained exposure, did not affect insulin sensitivity, as assessed using hyperinsulinaemic–euglycaemic clamp techniques [39, 40].

Limitations in the current study should be noted. First, metformin was administered as a solution for immediate release in the small intestine, and as such, our observations may be of less relevance to the use of extended- or delayed-release formulations. Second, to demonstrate proof-of-concept, we used a nasoduodenal catheter for administration of metformin. This model, while allowing precise control of the timing of small intestinal exposure to metformin and mitigation of the wide inter-individual variation in the rate of gastric emptying [29, 30], is somewhat ‘unphysiological’. Moreover, metformin is known to slow gastric emptying [8], which would contribute to reduction of postprandial glycaemia. It is therefore plausible that glucose-lowering by metformin may be more evident when glucose is administered orally. We used a glucose solution, instead of mixed nutrients, to minimise variations in digestion. Thus, it remains to be determined whether varying the timing of metformin administration affects postprandial handling of fat and protein. Third, we included a small amount of non-metabolisable glucose analogue, 3-*O*-methylglucose, in the glucose solution with the intention of measuring serum 3-*O*-methylglucose concentrations as an index of small intestinal glucose absorption. However, due to logistical issues, we were unable to perform these assays. For the same reason, plasma glucagon levels and metformin concentrations were not measured. However, the latter are unlikely to affect interpretation of the main conclusions, as administration of metformin by the enteral route has been shown to be more effective for glucose-lowering than intravenous or intraportal administration [5], and pharmacological inhibition of renal excretion of metformin, thereby augmenting plasma metformin concentrations, has little impact on blood glucose [41]. Fourthly, the sex distribution of the participants was unintentionally unbalanced in the current study. In view of recent evidence supporting a sex-driven disparity in the GLP-1 response to small intestinal glucose infusion [42], we cannot rule out the possibility of a sex-related difference in the effects reported. Finally, our study examined the impact of the time of metformin administration on postprandial glucose metabolism in an acute setting. Given our observations, further studies of longer duration are warranted to clarify whether administration of metformin 30–60 min before meals could improve postprandial and overall glycaemic control in type 2 diabetes.

In summary, in metformin-treated patients with well-controlled type 2 diabetes, metformin is more effective in glucose-lowering when given before, rather than with, enteral glucose, and this is associated with a greater GLP-1 response. These observations suggest that administration of metformin before meals may improve its capacity to improve postprandial glycaemic control.

## Data Availability

The datasets generated during and/or analysed during the current study are not publicly available but are available from the corresponding author on reasonable request.

## Abbreviations

GLP-1: Glucagon-like peptide-1
iAUC: Integrated AUC
Met: Metformin
